# Recent Advances in Hollow Gold Nanostructures for Biomedical Applications

**DOI:** 10.3389/fchem.2021.699284

**Published:** 2021-06-08

**Authors:** Jeong-Min Park, Hye Eun Choi, Dauletkerey Kudaibergen, Jae-Hyuk Kim, Ki Su Kim

**Affiliations:** ^1^ Department of Chemical and Environmental Engineering, Pusan National University, Busan, South Korea; ^2^ School of Chemical Engineering, Pusan National University, Busan, South Korea

**Keywords:** localized surface plasmon resonance, hollow gold nanostructures, biomedical application, biosensors, bioimaging, photothermal therapy

## Abstract

The localized surface plasmon resonance of metallic nanoparticles has attracted much attention owing to its unique characteristics, including the enhancement of signals in sensors and photothermal effects. In particular, hollow gold nanostructures are highly promising for practical applications, with significant advantages being found in their material properties and structures: 1) the interaction between the outer surface plasmon mode and inner cavity mode leads to a greater resonance, allowing it to absorb near-infrared light, which can readily penetrate tissue; 2) it has anti-corrosiveness and good biocompatibility, which makes it suitable for biomedical applications; 3) it shows a reduced net density and large surface area, allowing the possibility of nanocarriers for drug delivery. In this review, we present information on the classification, characteristics, and synthetic methods of hollow gold nanostructures; discuss the recent advances in hollow gold nanostructures in biomedical applications, including biosensing, bioimaging, photothermal therapy, and drug delivery; and report on the existing challenges and prospects for hollow gold nanostructures.

## Introduction

**FIGURE 1:**
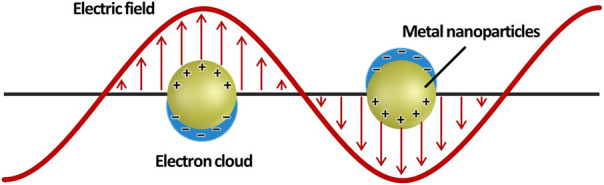
Schematic illustration of a metal nanoparticle’s electron cloud oscillation.

Owing to recent advances in the fabrication and analysis of metal nanoparticles, localized surface plasmon resonance (LSPR) has been extensively investigated and used in a broad range of fields. LSPR is the strong oscillation of the electron cloud of metal nanoparticles induced by incident light (Figure 1). This phenomenon increases the intensity of the electromagnetic field around the metal nanoparticles and is observed as a resonance absorption or scattering peak in the spectrum (Maier, 2007; Kreibig and Vollmer, 2013). The properties of LSPR can be affected by several factors, including the size and morphology of the nanoparticles, as well as the local dielectric environment (Jensen et al., 2000; Tran et al., 2020; Sekhon, 2021). By varying these factors, the LSPR peak can be tuned between visible and near-infrared light. The above-mentioned characteristics of LSPR allow us to apply it to various biomedical applications, such as biosensing (Lee et al., 2011), bioimaging (Feng et al., 2015), photothermal therapy (PTT) (Ding et al., 2014), and drug delivery (Bao et al., 2016). Gold nanostructures, a candidate material for effective LSPR, have been widely researched for their biomedical applications because of their large surface-to-volume (S/V) ratio, biocompatibility, and ease of surface modification (Chithrani et al., 2006; Lim et al., 2011). Gold nanostructures can be classified into two groups according to their structural symmetry: 1) isotropic, such as gold nanospheres, and 2) anisotropic, such as gold nanorods, nanoprisms, and nanostars. These gold nanostructures have unique optical and physical properties.

Among the aforementioned gold nanostructures, hollow gold nanostructures, which have hollow cavities inside them, have been intensively investigated owing to their unique advantages over pre-existing solid gold nanostructures (e.g., larger S/V ratio, enhanced resonance with the interaction of outer–inner plasmons, and availability of internal cavities to encapsulate other substances, which have the potential to be exploited in various biomedical applications) (Skrabalak et al., 2007; Goodman et al., 2014). Herein, we summarize the classification, characteristics, and synthetic methods of hollow gold nanostructures by focusing on their advantages and potential. The research trends and prospects of hollow gold nanostructures in biomedical applications corresponding to biosensors, bioimaging, PTT, and drug delivery are presented.

## Characteristics and Synthesis of Hollow Gold Nanostructures

### Characteristics of Hollow Gold Nanostructures

**FIGURE 2:**
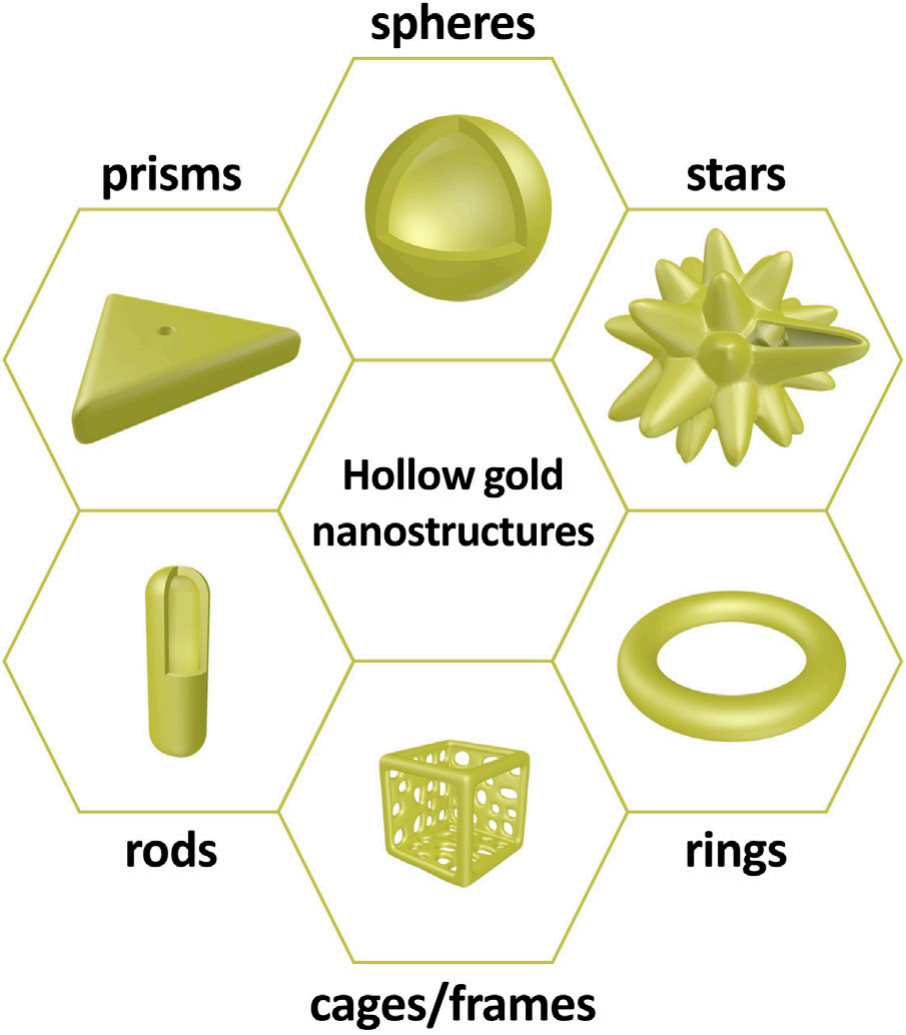
Schematic illustrations of hollow gold nanostructures: spheres, stars, rings, cages/frames, rods, and prisms.

Similar to conventional gold nanostructures, hollow gold nanostructures are promising materials for biomedical applications, owing to their chemical stability, biocompatibility, and ease of surface modification. However, the most apparent difference between solid and hollow gold nanostructures is the existence of an inner cavity. The inner cavity provides additional functionalities to hollow gold nanostructures, such as anticancer drug loading and interaction between the inner and outer surfaces; thus, these structures can be utilized as a new strategy for chemo–photothermal therapy or light-responsive drug release systems. Hollow gold nanostructures can be synthesized with various sizes and morphologies (including hollow gold nanospheres, gold nanocages/frames, gold nanorings, hollow gold nanostars, hollow gold nanorods, and hollow gold nanoprisms) depending on several factors, including the synthetic protocol, reaction temperature, and reaction atmosphere (Figure 2). Although there are differences in morphology, these hollow gold nanostructures have a thin gold shell in common, and the LSPR characteristics can be tuned by controlling the shell properties, which is not the case for solid gold nanostructures.

**FIGURE 3:**
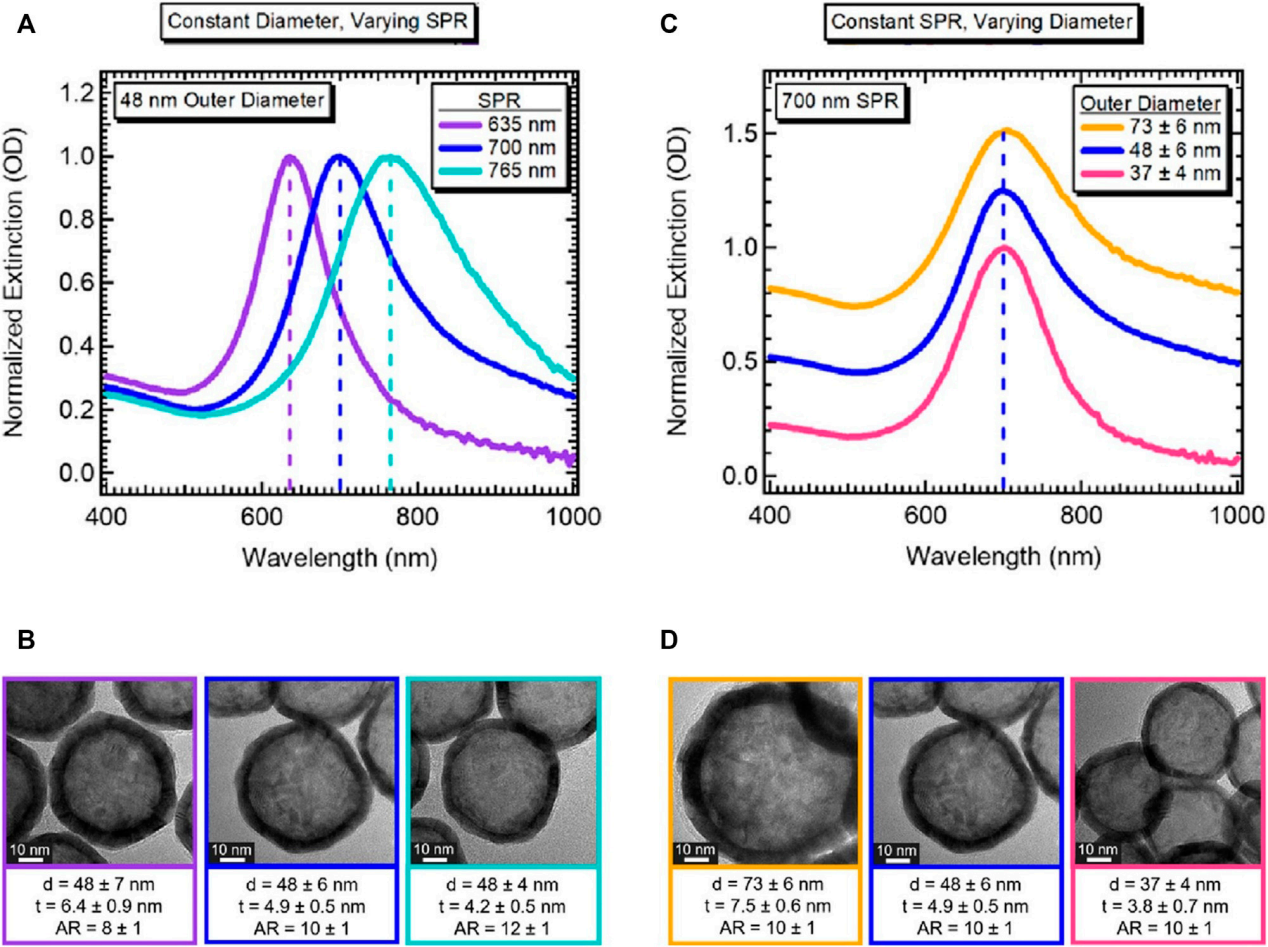
Demonstration of tunability of hollow gold nanospheres. **(A)** Normalized extinction for hollow gold nanospheres with the same outer diameter but different LSPR peaks. **(B)** Corresponding high-resolution transmission electron microscope (HRTEM) images with an average diameter (d), shell thickness (t), and aspect ratio (AR), as indicated; scale bar 10 nm. **(C)** Normalized extinction for hollow gold nanospheres with different outer diameters but the same LSPR peak. **(D)** Corresponding HRTEM images with *d*, *t*, and AR values as indicated (scale bar 10 nm). Reprinted with permission from Lindley et al. (2018). Copyright 2018, American Chemical Society.

Lindley et al. (2018) reported an improved size control method for Co_2_B nanoparticles and the role of environmental oxygen for galvanic exchange, which gives two-fold tunability of hollow gold nanospheres. They synthesized two types of hollow gold nanospheres with different diameters and shell thicknesses: 1) identical outer diameters with different aspect ratios (the ratio of outer diameter to shell thickness), providing LSPR peak tunability (Figures 3A,B), and 2) different outer diameters with the same aspect ratio, providing identical LSPR peaks that allow a range of hollow gold nanoparticles to have uniform energy distributions (Figures 3C,D). Specifically, this two-fold tunability is caused by the hybridization of plasmons, which is the interaction between the outer- and cavity-surface plasmons. Prodan et al. (2003) reported the highly geometry-dependent plasmon response of hollow gold nanospheres based on the hybridization of plasmons. The outer and inner plasmons interact with each other owing to the finite thickness of the hollow gold nanosphere shell; therefore, this interaction can be controlled by changing the thickness. The above-mentioned results show that hollow gold nanostructures provide an additional factor (thickness of the shell layer) to control the LSPR properties, and these hollow structures are more viable than conventional solid gold nanostructures for various applications that require a certain particle size or specific LSPR peaks.

### Synthesis of Hollow Gold Nanostructures

#### Metal-Template-Based Synthesis (Galvanic Replacement)

**FIGURE 4:**
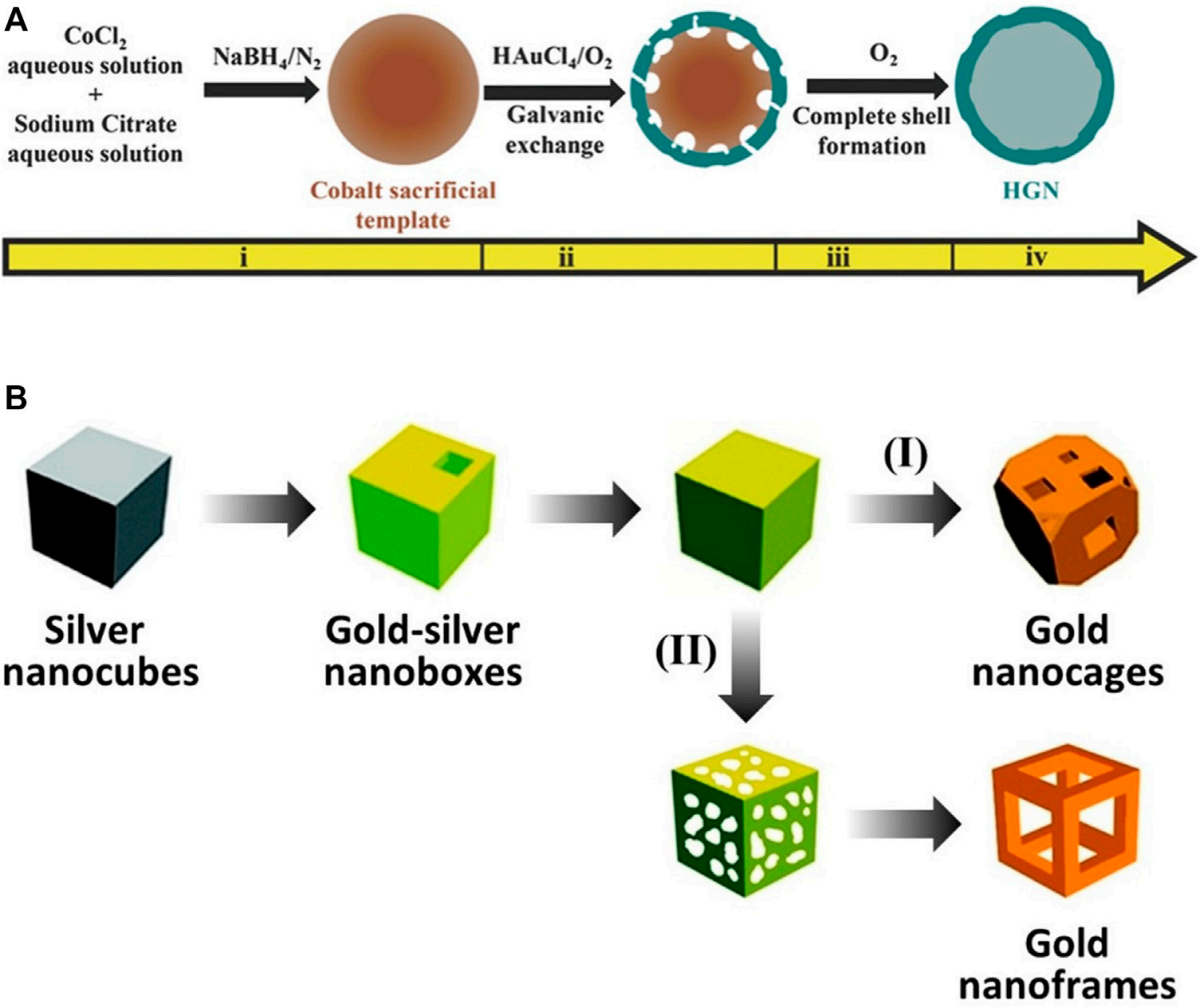
Schematic illustration of the synthesis of hollow gold nanostructures based on the galvanic replacement method. **(A)** Hollow gold nanospheres using cobalt nanospheres as sacrificial templates. **(B)** Gold nanocages/frames using silver nanocubes as sacrificial templates. **(A)** Reprinted with permission from Pu et al. (2017). Copyright 2016, WILEY-VCH Verlag GmbH and Co. KGaA, Weinheim. **(B)** Reprinted with permission from Skrabalak et al. (2008). Copyright 2008, American Chemical Society.

Hollow gold nanostructures—even the same structures—have been synthesized by various methods; however, synthesis using galvanic replacement is significantly dominant (Xia et al., 2013). Galvanic replacement is driven by the difference in electrochemical potential between the solid metal (regarded as A), which has a lower reduction potential, and another metal ion (regarded as B^+^), which has a higher reduction potential. While the solid metal with the lower potential is oxidized and transformed into a metal ion (from A to A^+^), the metal ion with the higher potential is reduced and transformed into a solid metal (from B^+^ to B) at the contact surface. Through these processes, hollow nanostructures can be successfully synthesized with several characteristics: 1) tunable elemental compositions (Lu et al., 2007b), 2) easily controllable porous shells (by varying the amounts of precursors for galvanic replacement) (Sun and Xia, 2004), 3) closely similar shapes to sacrificial templates (Chen et al., 2006), 4) short reaction time (∼30 min) (Cobley and Xia, 2010), and 5) high S/V ratio (Oh et al., 2013). For hollow gold nanospheres and gold nanocages/frames, the typical synthetic methods use cobalt nanoparticles and silver nanocubes as sacrificial templates, respectively (Figures 4A,B). Liang et al. (2005) reported uniformly synthesized hollow gold nanospheres using cobalt nanoparticles as sacrificial templates for galvanic replacement; this synthetic method, with minor modifications, has been widely used to fabricate hollow gold nanospheres. The cobalt nanoparticle sacrificial templates were first synthesized by reducing Co^2+^ with NaBH_4_ under a nitrogen atmosphere to avoid oxidation. The hollow gold nanostructures were then fabricated as Au^+^ and reduced onto the cobalt nanoparticles based on galvanic replacement and finally stirred under ambient conditions to ensure complete oxidation of the residual cobalt nanoparticles. The shape of the final hollow gold nanostructure depends on the shape of the sacrificial template employed, as well as the post-treatment processes. Sun and Xia (2004) and Lu et al. (2007a) reported gold nanocages and gold nanoframes using silver nanocubes as a sacrificial template. In this process, gold-silver nanoboxes are first fabricated as HAuCl_4_ and reduced onto the silver nanocubes, then gold nanocages or gold nanoframes are synthesized depending on the following processes: 1) further galvanic replacement by HAuCl_4_ (path [I] in Figure 4B, gold nanocages) or 2) selective removal of silver using wet etchants such as Fe(NO_3_)_3_ and NH_4_OH (path [II] in Figure 4B, gold nanoframes).

**FIGURE 5:**
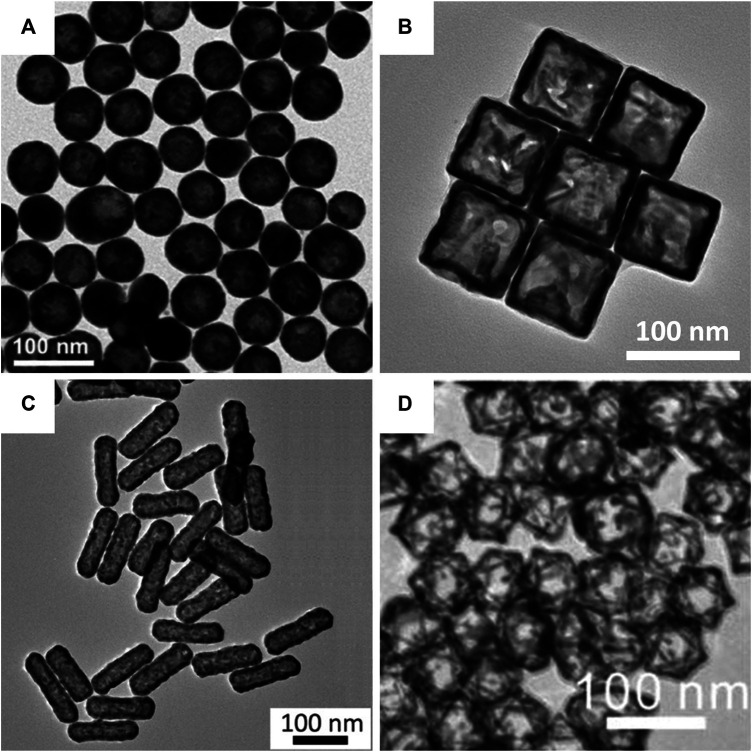
Transmission electron microscopy (TEM) images of hollow gold nanostructures based on the galvanic replacement method. **(A)** Hollow gold nanospheres. **(B)** Hollow gold nanocages. **(C)** Hollow gold nanorods. **(D)** Hollow gold octahedral nanoframes. **(A)** Reprinted with permission from Choi et al. (2020). Copyright 2020, Elsevier. **(B)** Reprinted with permission from Wang W. et al. (2015). Copyright 2015, Royal Society of Chemistry. **(C)** Reprinted with permission from Cai et al. (2018). Copyright 2018, American Chemical Society. **(D)** Reprinted with permission from Hong et al. (2012). Copyright 2012, American Chemical Society.

The hollow structures were confirmed using transmission electron microscopy (TEM). In addition to nanospheres (Figure 5A; cobalt nanoparticle-templated) and nanocages (Figure 5B; silver nanocube-templated), more complex hollow gold nanostructures, such as nanorods (Figure 5C; tellurium nanorod-templated) and octahedral nanoframes (Figure 5D; silver nanoparticle-templated), were successfully synthesized with uniform size and morphology by galvanic replacement. Unlike hollow gold nanospheres and nanocages/frames, hollow gold nanorods are fabricated using a new metallic sacrificial template; tellurium nanorods (Cai et al., 2018). They reported the synthesis of hollow gold nanorods with controllable aspect ratios using a selenium-doped tellurium nanorod-templated method for the first time. In addition to the hollow gold nanostructures in Figure 5, other hollow gold nanostructures, including nanostars (Zhu et al., 2020; cooper nanoparticle-templated), nanorings (Prieto et al., 2014; cobalt nanoparticle-templated), and nanoprisms (Aherne et al., 2010; silver nanoprism-templated) can also be synthesized by galvanic replacement. It is worth noting again that galvanic replacement is the major approach for the synthesis of hollow gold nanostructures.

#### Non-galvanic Replacement-Based Synthetic Methods

Although galvanic replacement is a major synthetic method for hollow gold nanostructures, there are some drawbacks, such as 1) fragmentation or aggregation due to excessive galvanic replacement processes (Lee et al., 2014) and 2) the generation of insoluble byproducts that can be deposited on the surface of nanoparticles (Zhang et al., 2012). To overcome these issues, several other approaches have been tested as alternatives to galvanic replacement.

**FIGURE 6:**
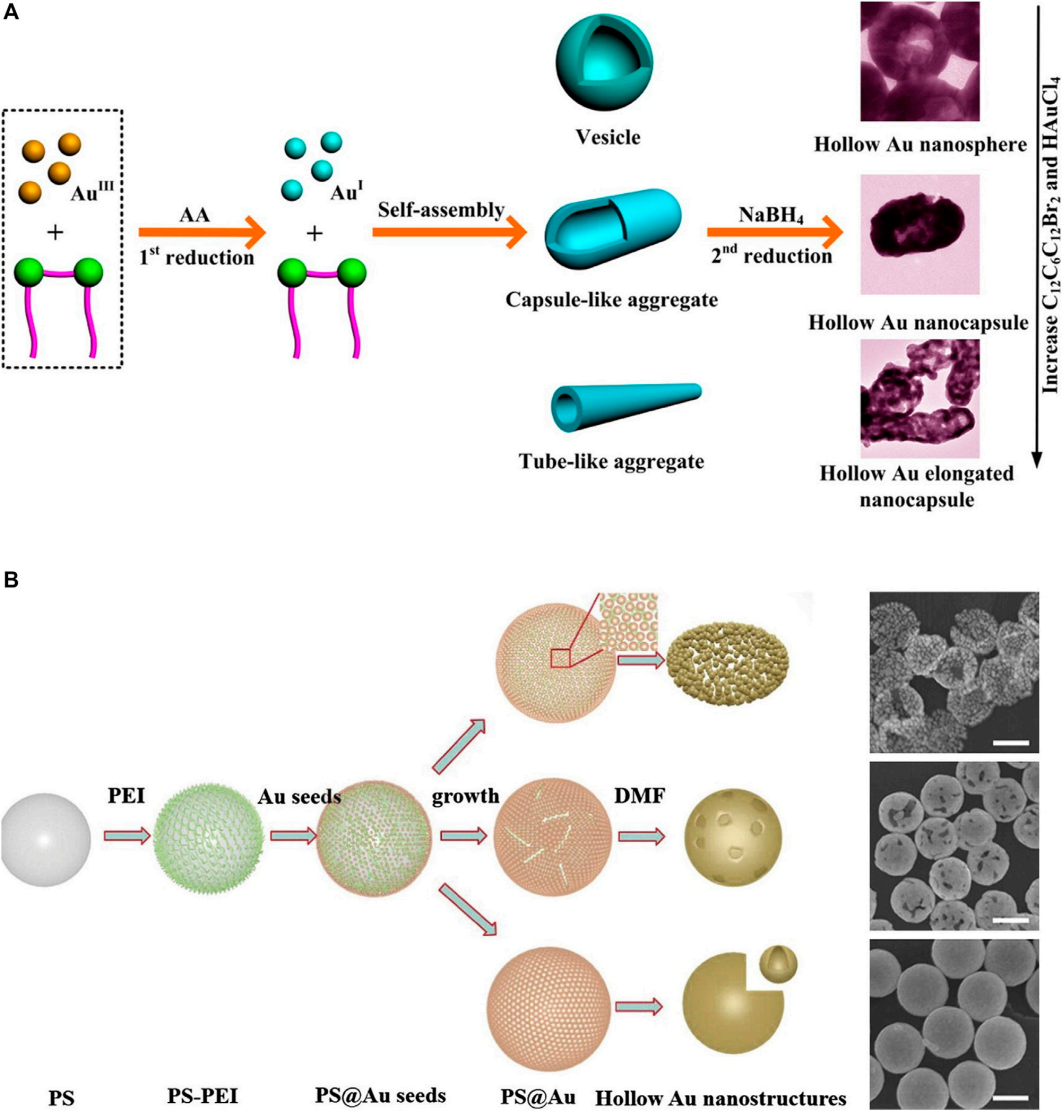
Schematic illustration of the synthesis of hollow gold nanostructures based on non-galvanic replacement method and its transmission electron microscopy (TEM) images. **(A)** Hollow gold nanostructure synthesis using the stepwise reduction method. **(B)** Hollow gold nanostructures from polystyrene (PS)@Au core–shell nanospheres, scale bar: 200 nm. **(A)** Reprinted with permission from Wang et al. (2013). Copyright 2013, American Chemical Society. **(B)** Reprinted with permission from Wang H. et al. (2015). Copyright 2014, Elsevier.

Wang et al. (2013) reported the successful fabrication of various hollow gold nanostructures, such as nanospheres, nanocapsules, and elongated nanocapsules, by employing a facile soft template approach (Figure 6A). Here, a cationic gemini surfactant, hexamethylene-1,6-bis(dodecyl dimethylammonium bromide) (C_12_C_6_C_12_Br_2_), played an important role as a soft template. C_12_C_6_C_12_Br_2_ is induced to self-assemble into various forms of vesicles, such as capsule-like or tube-like aggregates, depending on its concentration. Moreover, it provides structural stability during reduction because of its strong aggregation tendency. Wang H. et al. (2015) reported the synthesis of gold nanocages and hollow gold nanospheres from polystyrene (PS)@Au core–shell nanospheres using solvent thermal treatment in N, N-dimethylformamide (DMF) (Figure 6B). Specifically, PS@Au core–shell nanospheres were synthesized by attaching gold seeds to PS surfaces, which were further modified with branched polyethyleneimine to enhance the absorption of gold seeds onto the PS surfaces. Then, gold nanocages and hollow gold nanospheres were fabricated by removing the PS templates of the PS@Au core–shell nanospheres with thermal treatment in DMF, and the morphologies were affected by the core–shell structures or the temperature and time of thermal treatment.

## Biomedical Applications of Hollow Gold Nanostructures

Recently, inert and relatively biocompatible gold nanostructures have been adopted for various biomedical applications owing to their attractive chemical and physical properties (Tiwari et al., 2011; Ren et al., 2015; Zangabad et al., 2017). Gold nanostructures have significant potential benefits in biomedical and industrial applications, although they can have harmful effects on biological systems such as bioaccumulation, bioclearance, metabolism, and elimination. To resolve these limitations, many studies have been conducted to mitigate and improve adverse influences through modifications including diameter, coating, shape, dosage, administration route. (Lopez-Chaves et al., 2018). Among these families of gold nanostructures, hollow gold nanostructures are one of the most promising platforms in biomedical applications owing to their unique characteristics (Ko et al., 2013; Han et al., 2018). They are characterized by compact sizes of approximately 20–500 nm, simple and precise LSPR adjustments, and large absorption cross-sectional area per unit volume. These characteristics allow hollow gold nanostructures to be mass-produced in high quality with the capacity to load and release drugs owing to their hollow interiors.

**FIGURE 7:**
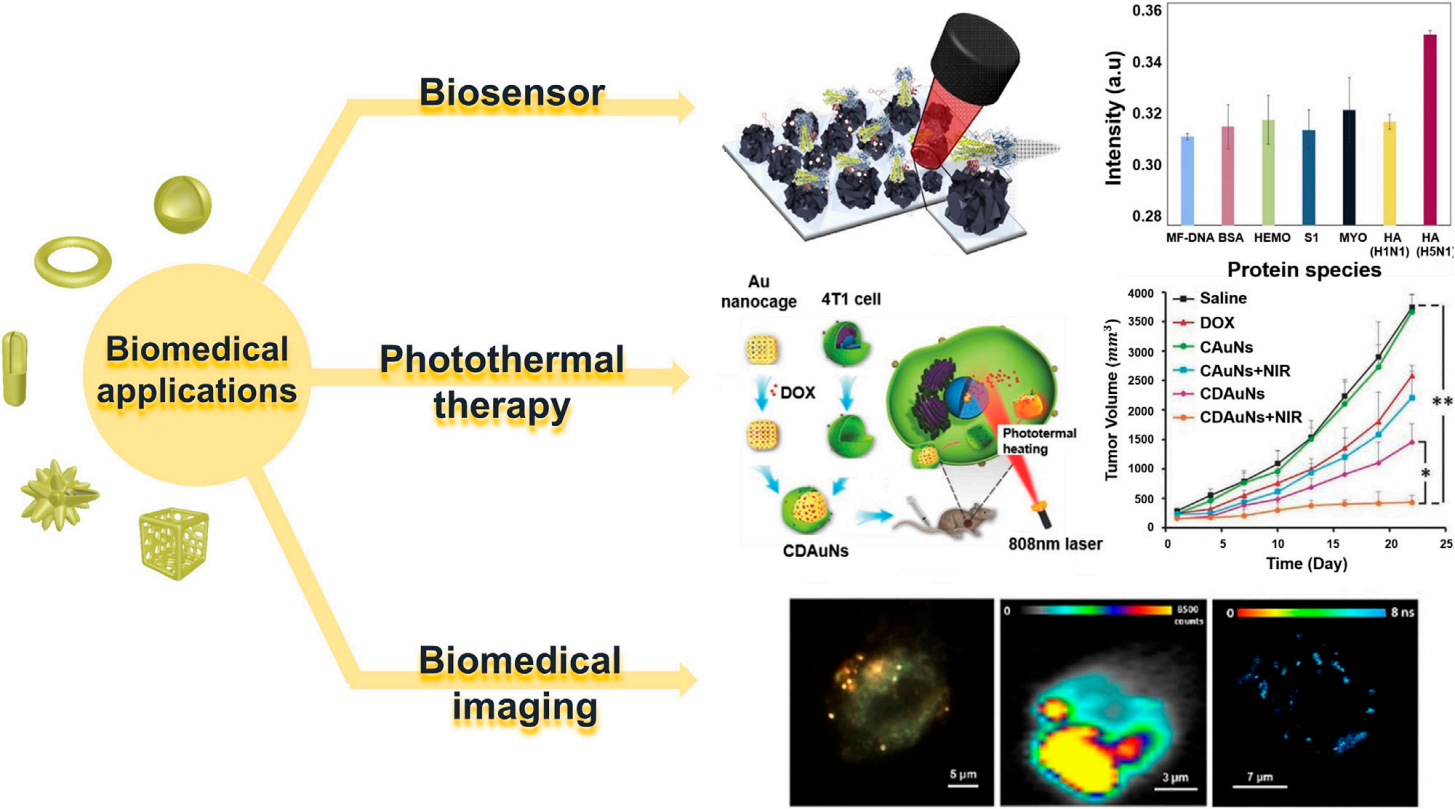
Various biomedical applications of hollow gold nanostructures. Reprinted with permission from Lee et al. (2019). Copyright 2018, Elsevier. Reprinted with permission from Sun et al. (2017). Copyright 2016, WILEY-VCH Verlag GmbH and Co. KGaA, Weinheim. Reprinted with permission from Nagy-Simon et al. (2017). Copyright 2017, American Chemical Society.

Hollow gold nanostructures are also viable in complex *in vivo* environments owing to their high mechanical flexibility and stability; they can be used as suitable platforms for a variety of purposes, such as labeling with various ligands, biomolecules, and functionalization because of their atomically flat surfaces (Satija et al., 2015; Ye et al., 2020). Table 1 briefly shows recent studies in hollow gold nanostructures that can be used in various biomedical applications. Therefore, in this section, we summarize three types of biomedical applications using hollow gold nanostructures: biosensors (diagnosis), PTT, and bioimaging applications (theragnostics) (Figure 7).

**TABLE 1:** Summary of recent studies in hollow gold nanostructures for biomedical applications.

	Structure type	Synthetic method	Sacrificial template	References
Biosensor	Sphere	Galvanic replacement	Co nanoparticle	Azadmehr and Zarei. (2020); Choi et al. (2020)
			Ag nanoparticle	Lee et al. (2019)
	Cage	Galvanic replacement	Ag nanocube	Chen et al. (2021)
PTT	Sphere	Galvanic replacement	Cobalt nanoparticle	Lindley and Zhang (2019); Shen et al. (2020)
			Ag nanoparticle	He et al. (2021)
	Cage	Galvanic replacement	Ag nanoparticle	Fang et al. (2020)
	Rod	Galvanic replacement	Te nanorod	Zhang et al. (2019)
	Star	Galvanic replacement	Ag-SiO2 nanoparticle	Kim et al. (2020)
		Galvanic replacement	Cu nanoparticle	Zhu et al. (2020)
Biomedical imaging	Sphere	Galvanic replacement	Co nanoparticle	Cui et al. (2020)
		Non-galvanic replacement	-	Depciuch et al. (2020)
	Cage	Galvanic replacement	Ag nanocube	Gao et al. (2020)
			Ag nanoparticle	Wang et al. (2020)
	Rod	Galvanic replacement	TeSe nanorod	Cai et al. (2020a)

### Biosensor: Diagnostics Application

Biosensors have been developed for various analyses across the fields of medicine, food testing, environmental sensing, and process control monitoring for research and industry. It is defined as a transducer that includes biological recognition components and consists of a three-factor system of bioreceptors, transducers, and signal processing devices (Conroy et al., 2009; Goode et al., 2015; Yoon et al., 2013). The distinct LSPR property is one of the most representative features of gold nanoparticles, and the peak position of LSPR is affected by the morphology, size, and surrounding medium of the nanoparticles; in particular, it is closely related to the refractive index (RI) of the material close to the nanoparticle surface. Because LSPR relies on RI around nanoparticles, it can be the basis for detecting interactions with molecules near nanoparticle surfaces (Ko et al., 2015; Zhang et al., 2018). The scattering spectrum of a single plasma resonance nanoparticle provides a single molecular event, and for nanoparticles, it can have a high RI sensitivity limited to nanometer-scale sensing volumes surrounding pointed tips. The LSPR-based biosensor uses a mechanism to observe noticeable changes in the peak wavelength of plasma resonance when a single giant molecule, such as a protein with a RI different from that of water, enters or leaves this sensing volume. Hollow gold nanostructures have higher RI sensitivities than solid gold nanostructures (Mayer et al., 2010; Ren et al., 2015; Zeng et al., 2020; Kim et al., 2020).

**FIGURE 8:**
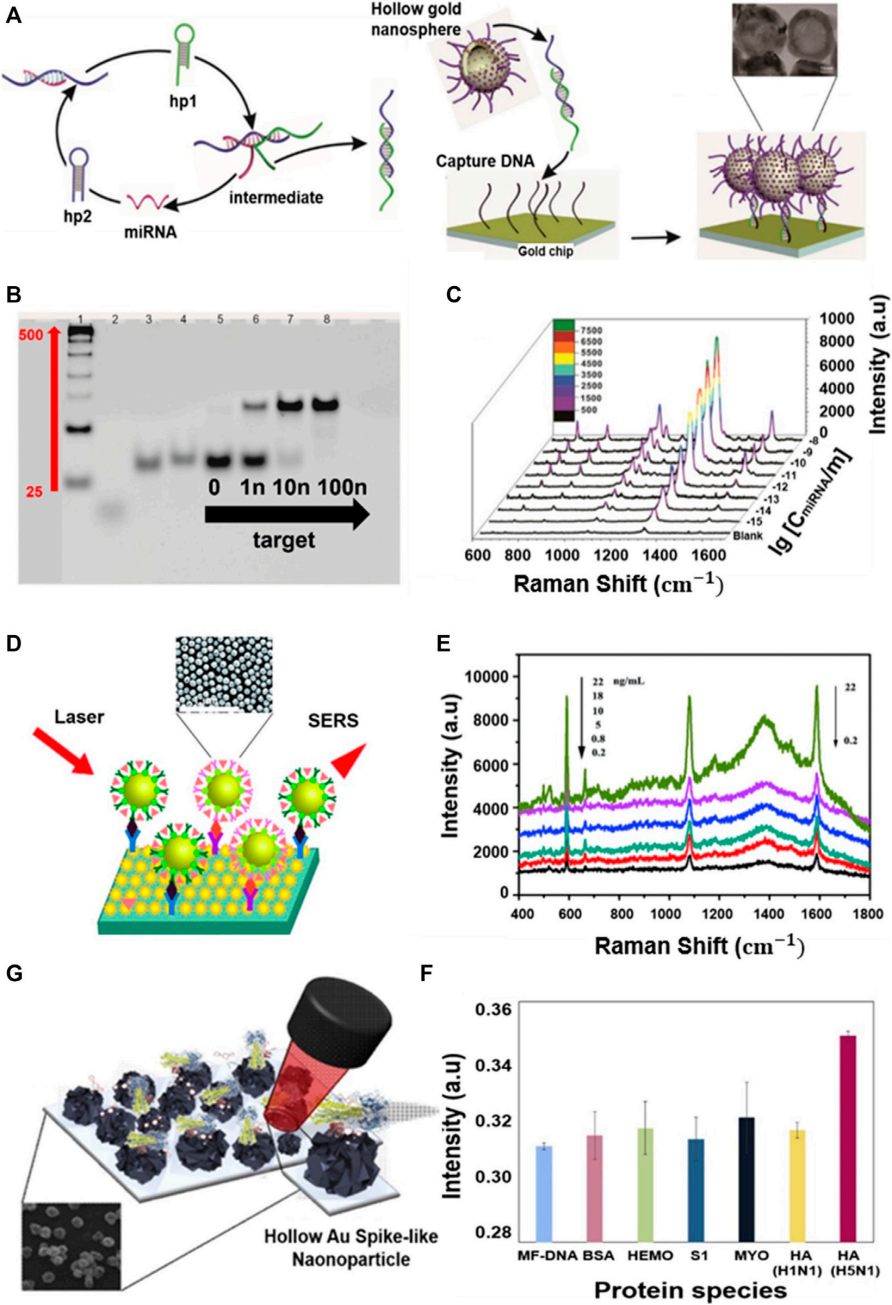
miRNA-detection biosensor for acute myocardial infarction diagnosis. **(A)** Target-catalyzed hairpin assembly process and final structure for the surface-enhanced Raman spectroscopy detection platform. **(B)** Polyacrylamide gel electrophoresis results of the miR-133a-powered DNA molecular machine. **(C)** Representative SERS spectra corresponding to miR-133a with varying concentrations. Biosensor to detect brain damage-related proteins. **(D)** Illustration of SERS-based immunoassay for neuron-specific enolase (NSE) and S100 calcium-binding protein beta (S100-β) detection. **(E)** SERS spectra of the multiplex immunosensor after incubation with various concentrations of S100-β and NSE. Biosensor utilizing DNA to detect avian influenza virus. **(F)** Schematic image of the fabricated AIV detection biosensor based on the LSPR method. **(G)** Change in absorbance peak based on selectivity test with other protein reactions: cytochrome c (blue line), BSA (green line), spike protein (gray line), myoglobin (yellow line), HA protein (H1N1) (purple line), and HA protein (H5N1) (red line). **(A, B, C)** Reprinted with permission from Sun and Li (2018). Copyright 2018, American Chemical Society. **(D, E)** Reprinted with permission from Wang et al. (2018). Copyright 2018, The Royal Society of Chemistry. **(F, G)** Reprinted with permission from Lee et al. (2019). Copyright 2018, Elsevier.

Detect of disease-related biomarkers plays an important role in the diagnosis and treatment. Among the various biosensors, surface-enhanced Raman spectroscopy (SERS)-based biosensor is one of the most powerful spectroscopic tools for biomedical field with high sensitivity and specific fingerprints and fast data collection speeds (Sutter et al., 2020; Cai et al., 2020b; Chung et al., 2015; Jeong et al., 2019). A hollow silver/gold nanosphere-based SERS probe was developed by Sun and Li (2018) for the diagnosis and treatment of acute myocardial infarction (AMI), a severe cardiovascular disease. This hollow nanostructure enables the ultra-high-sensitivity detection of AMI-related miRNA (miR-133a) using a target-catalyzed hairpin strategy. In this process, the target miRNA was used directly as a linker to capture SERS-based probes in sandwich mode, and a duplex linker with two sticky ends was selected in the target catalyst hairpin assembly strategy (Figures 8A–C). Polyacrylamide electrophoresis experiments were then performed to verify that hairpin DNA 1 and 2 (hp1 and hp2) were consumed to generate targets in SERS analysis and that miR-133a was used as a linker to hold probes on the substrate. Consequently, the presence of miR-133a became increasingly apparent as the new band moved slowly and increased in concentration, and the hp1, hp2 mixture disappeared from lanes to 6–8 (Figure 8B). In the SERS spectrum of miR-133a at various concentrations, the strength of the peak appeared to increase with increasing Raman dye (nitroblue tetrazolium) concentrations (Figure 8C) (Sun and Li, 2018).

For protein detection, an immune sensor to diagnose brain damage by detecting neuron-specific enolase (NSE) and S100-b proteins in the blood was developed (Wang et al., 2018). The active group of hollow gold nanospheres and the redox molecules 4-mercaptobenzoic acid (4-MBA) and Nile blue A incorporate antibodies and provide signals to design a simple label-free three-dimensional hierarchical plasmonic nanostructure (Figures 8D,E). In the presence of protein biomarkers, sandwich nanoparticles are captured on a substrate to develop a finite plasma field that amplifies Raman signals. Thus, as the S100-b and NSE protein concentrations increased, the peaks in the SERS spectrum became stronger (Figure 8E). In the case of hollow gold spike-like nanoparticles, a biosensor was also reported to detect avian influenza virus (AVI) with the introduction of a multi-functional DNA 3 way-junction (Figures 8F,G) (Lee et al., 2019). To detect AVI, each piece of the DNA 3 way-junction was adjusted to a hemagglutinin (HA) binding aptamer, 5′-fluorescein phosphoramidite (FAM) dye, or thiol group, respectively. In this system, the hollow gold spike-like gold nanoparticles were fixed to the ITO substrate to measure LSPR and immobilized onto the electrode *via* the thiol group. The fabricated biosensor detected HA proteins in phosphate-buffered saline (limit of detection (LOD): 1 pM) and diluted chicken serum (LOD: 1 pM) and confirmed that the LSPR peak increased as the concentration increased from 1 pM to 100 nM (Figure 8G).

### Photothermal Therapy: Therapeutic Application

PTT is an effective cancer treatment method using nanoparticles that utilize a mechanism in which light is absorbed by nanoparticles and converted into heat within tumor tissues. When the local temperature in tumor tissue reaches 42–47°C, tumor cells—owing to their heat sensitivity being higher than that of normal cells—quickly undergo necrosis (Farokhnezhad and Esmaeilzadeh, 2019). This treatment is characterized by minimal invasiveness and excellent tissue penetration. Gold nanoparticles have received much attention because of their unique photothermal conversion properties and LSPR properties (Bi et al., 2019; Ling et al., 2021). The high affinity of tumor cells for the applied gold nanoparticles should have an efficient photothermal effect, so appropriate receptors or ligands should be modified on the particle surface to enable tumor-specific targeting (Grabowska-Jadach et al., 2019; Xu et al., 2021).

**FIGURE 9:**
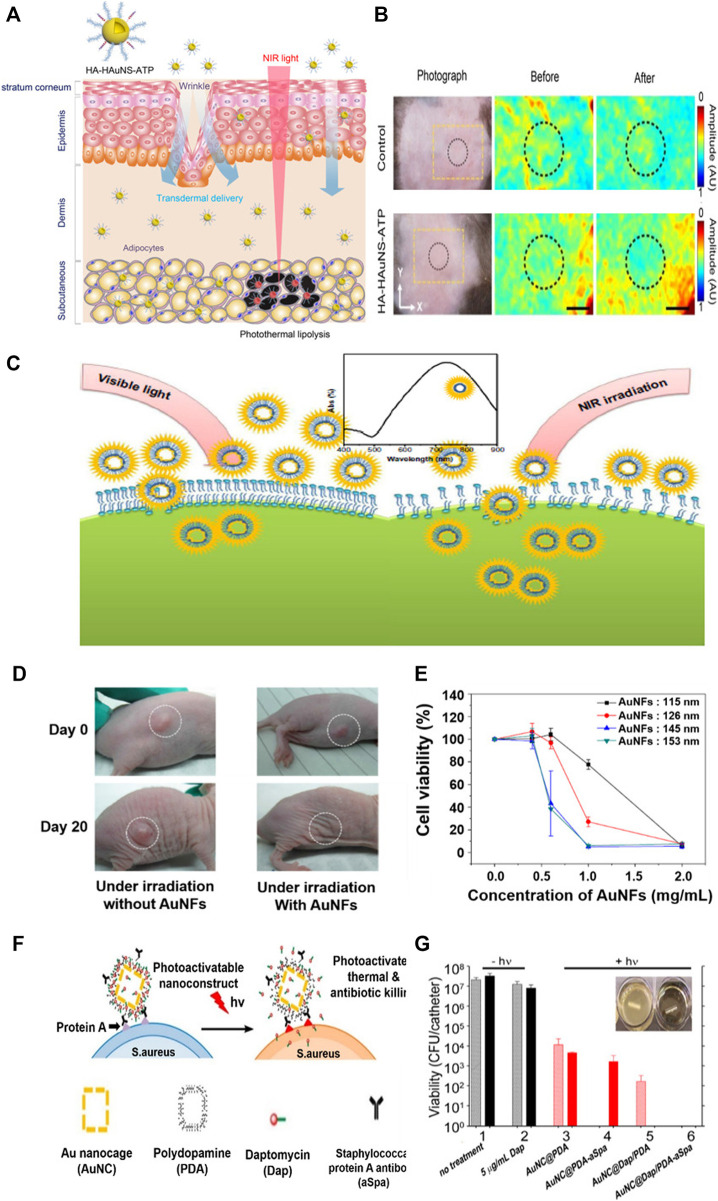
Transdermal hyaluronic acid-hollow gold nanosphere-ATP conjugate for obesity treatment. **(A)** Schematic illustration of the targeted photothermal lipolysis of adipocytes after noninvasive transdermal delivery. **(B)** Photoacoustic images of subcutaneous adipose tissues before and after NIR lasers. Hollow gold nanoflowers with strong photothermal effects in near-infrared (NIR) areas. **(C)** Schematic representation showing the photothermal effect of the gold nanoflowers (AuNFs) on endocytosis under NIR irradiation. **(D)** Photothermal effect on a nude mouse under NIR irradiation caused by the addition of gold nanoflowers. **(E)** Relationship between HeLa cell viabilities and the concentration of gold nanoflowers with laser irradiation (λ = 808 nm, 9 W/cm^2^, 5 min). Gold nanocages (AuNC) loaded with antibiotics and coated with polydopamines (PDAs) to induce bacterial apoptosis. **(F)** Schematic illustration of the working mechanism of the targeted photoactivatable nanostructure for the synergistic photothermal and antibiotic treatment of *S. aureus*. **(G)** Bacterial cell killing using a biofilm model. Experimental groups are 1) no treatment, 2) 5 μg/ml Dap, and irradiation plus 3) AuNC@PDA, 4) AuNC@PDA−aSpa, 5) AuNC@DapHi/PDA, and 6) AuNC@DapHi/PDA−aSpa. Killing was assessed at 0 h (striped bars) and 24 h (solid bars) after treatment. Black bars indicate non-irradiated groups, and red bars indicate irradiated groups. **(A, B)** Reprinted with permission from Lee et al. (2017). Copyright 2017, American Chemical Society. **(C, D, E)** Reprinted with permission from Han et al. (2014). Copyright 2014, Dovepress. **(F, G)** Reprinted with permission from Meeker et al. (2016). Copyright 2016, American Chemical Society.

Gold nanostructures can be used to treat obesity. Their easy facile modification, superior stability, nontoxicity and biocompatibility make this possible. In addition, the properties of absorbing visible and near-infrared light to convert to thermal energy lead to effective photothermal treatment. Figures 9A,B achieves non-invasive photothermal ablation targeting abnormal adipose tissue through hyaluronic acid and peptides, and hollow gold nanosphere (Lee et al., 2017). This is transmitted through the subcutaneous, efficiently targeted at adipocytes, and enables highly effective photothermal ablation with NIR laser. The successful results were reported in Figure 9B through experiments in C57BL/6 obesity mice. After topical treatment of hollow gold nanosphere conjugated with hyaluronic acid and ATP in the abdominal region of mice, NIR lasers were investigated for photothermal lipolysis for 10 min. As a result, the significant reduction of photoacoustic signals in adipose tissue in the dotted area confirmed effective photothermal lipolysis, and no side effects were shown.

One of the advantages of hollow gold nanostructure is that it is easy to control LSPR, which can be adjusted in size of nanostructure from the visible light field to the near-infrared field for cancer photothermal therapy. Figures 9C–E shows a study that produced a hollow gold nanoflower structure to indicate high biological stability in visible light and very strong photothermal cytotoxicity to tumor cells in near-infrared environments (Han et al., 2014). Experiments were conducted with hollow gold nanoflowers using surfactant bis-(amidoethyl-carbamoylethyl) octadecylamine (C18N3) as templates with multi-amine head groups on a tumor-bearing mouse model. Therefore, tumor size increased in the control group. However, the tumor almost disappeared from the lesion after 20 days in the gold nanoflower-treated mice (Figure 9B). Figure 9E shows a substantial decrease in HeLa cell viability affected by 808 nm NIR laser, size, and concentration of gold nanoflowers.

Hollow gold nanostructures can be used to treat bacterial infections and cancers. Studies have demonstrated the synergistic treatment of bacterial infections by loading antibiotic daptomycin (Dap) into polydopamine (PDA)-coated gold nanocages (Figures 9F,G) (Meeker et al., 2016). To induce target binding to bacterial cell surface proteins (Spa) *via* anti-Spa, PDA was coated onto the surface and the nanostructure was activated by near-infrared radiation to convert photon energy into thermal energy, increasing the photothermal effect and degenerating the PDA coating to release the antibiotics (Figure 9F). Figure 9G shows the results of antibiotic treatment on 0.5 cm segments of catheter biofilm. Exposure to 5 μg/ml Dap in groups 1 and 2 had a slight effect on the number of viable bacteria per catheter compared to that in the control group, where nothing was processed. Exposure to untargeted gold nanocage@PDA after laser irradiation showed an insignificant decrease in the number of viable bacteria per catheter from 0 to 24 h (Group 3). With aSpa, the photothermal effect was increased immediately after irradiation on targeted gold nanocage@PDA, but it can be seen to increase from 24 h because of its failure to completely exterminate viable bacteria in the biofilm (Group 4). In group 5, a clear photothermal effect was observed shortly after laser irradiation of bacteria exposed to gold nanocage@ $\mathrm{Da}p_{Hi}/PDA-aSpa$, and no viable bacteria were detected at 24 h. In addition, bacteria were not detected at either 0 or 24 h when the biofilm was laser-irradiated by exposure to gold nanocage@ $\mathrm{Da}p_{Hi}/PDA-aSpa$. These results confirmed the synergistic effects of photothermal killing and targeted antibiotic release.

Hollow gold nanostructures have high photothermal conversion effects and excellent biocompatibility, can adjust the LSPR band in near-infrared (NIR) regions, and are suitable for drug delivery. The irradiation of gold nanoparticles with NIR lasers can accelerate the release of cargo by generated hyperthermia to the target location and ablate the tumor cells with favorable PTT (Gan et al., 2019; Sun et al., 2021).

**FIGURE 10:**
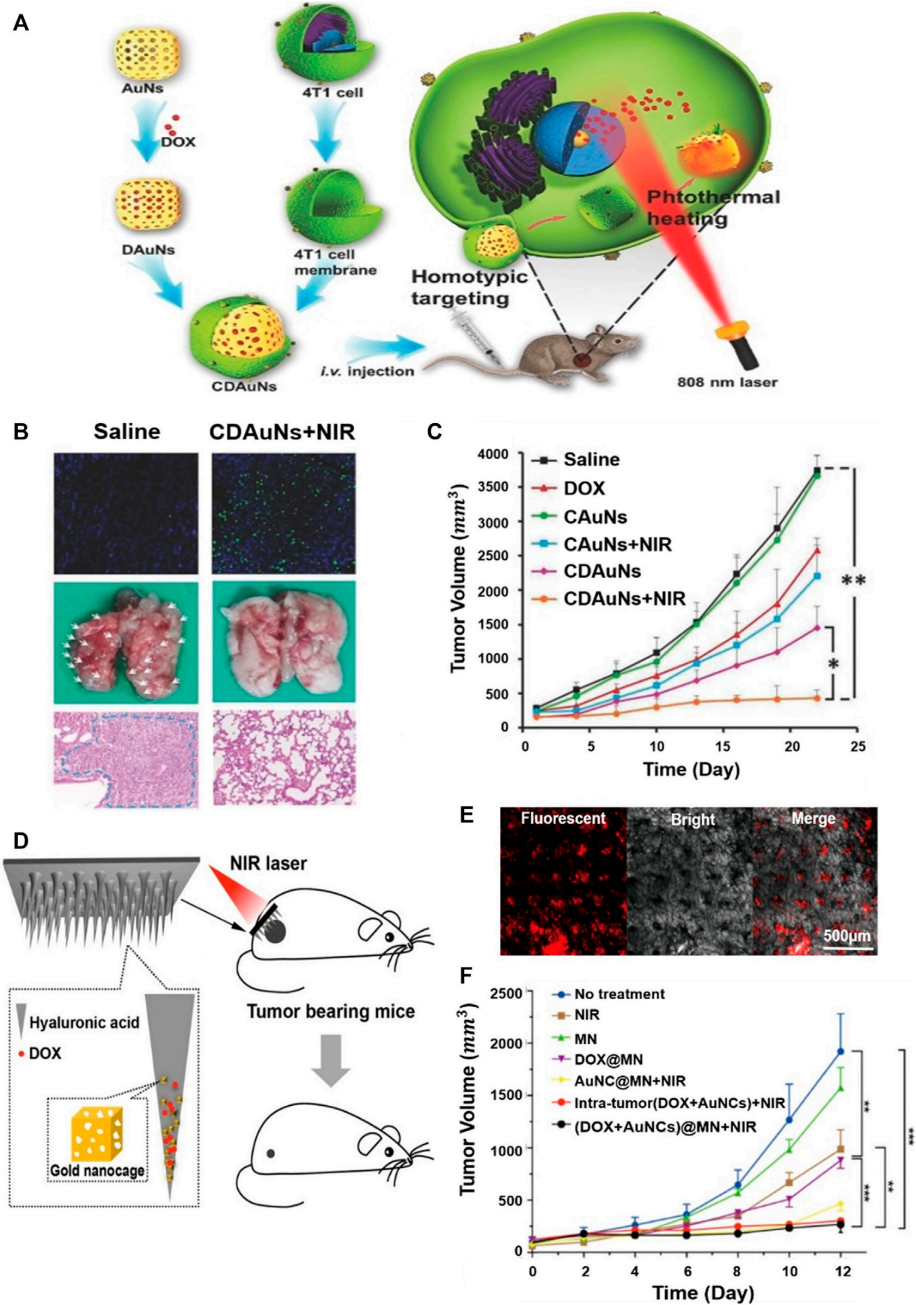
Gold nanocages that suppress high breast cancer metastasis by coating cancer cell membranes. **(A)** Schematic illustration of the 4T1 cancer cell membrane-coated gold nanocage for the hyperthermia-triggered release of doxorubicin (DOX) and homotypic targeted therapy of breast tumor growth and metastasis. **(B)** Terminal deoxynucleotidyl transferase (TdT)-mediated dUTP nick-end labeling (TUNEL) staining (200×) of the tumor tissues, the collected lungs, and H&E staining (Scale bar = 100 µm) of the lung tissue. White arrows indicate the metastatic models. **(C)** *In vivo* antitumor and antimetastasis effects of the nanoparticles in the 4T1 tumor-bearing nude mice of tumor growth profiles. Example of applying gold nanocages to microneedles for the local treatment of superficial skin tumors. **(D)** Schematic illustration showing drug/gold nanocage (AuNC)-loaded dissolving hyaluronic acid microneedle (HA MN) system for the combined chemotherapy and PTT of melanoma. **(E)** Confocal laser scanning microscope (CLSM) image, bright-field dermoscopy image, and merged image. The red fluorescence signal in the CLSM image indicated the penetration of AuNCs in the skin indicated, whereas the black dots in the bright-field image indicated the MN insertion sites. **(F)** Tumor volume of each group with increasing time for 12 days **(A, B, C)** Reprinted with permission from Sun et al. (2017). Copyright 2016, WILEY-VCH Verlag GmbH and Co. KGaA, Weinheim. **(D, E, F)** Reprinted with permission from Dong et al. (2018). Copyright 2018, American Chemical Society.

By combining PTT with encapsulating drugs in the internal space of hollow gold nanostructures where LSPR control is easy, improved therapeutic results can be achieved compared to the existing method. But the innate structures of the hollow gold nanostructure can leak drugs early, which makes decreases targeting efficiency. To prevent this, utilizing biomimetic cell membrane-based drug delivery systems can significantly increase the stability of nanoparticles and prevent drug leakage. Research has been conducted using cancer cell membranes coated with gold nanocages to suppress breast cancer metastasis (Figures 10A–C) (Sun et al., 2017). In chemotherapy, nanostructures coated with biomimetic cell membranes can significantly increase stability with fewer drug leaks under physiological conditions. Light heat at 43°C can also increase the permeability and fluidity of the cell membrane, thereby improving the intracellular accumulation of drugs. *Cancer* cell membrane-coated gold nanocages were fabricated by loading the anticancer drug Doxorubicin (DOX) into the inner core of gold nanocages and then coating with the cancer cell membrane derived from 4T1 breast cancer cells. A sulforhodamine B assay to determine the antiproliferative effect of gold nanocages showed that the strongest antiproliferative effect was on tumor cells cultured with cancer cell membrane-coated gold nanocages, resulting from the accelerated release of DOX from gold nanocages by laser-induced high heat and induced phototoxicity (Figure 10B). The *in vivo* inhibitory effect of cancer cell membrane-coated gold nanocages on tumors and pulmonary metastasis is shown in Figure 10C. Evaluation of the 4T1 tumor transition model showed rapid tumor growth in the saline treatment group and excellent antitumor effects in the NIR investigation group.

A minimally invasive transdermal drug delivery system is available to minimize the side effects and pains (Li et al., 2017; Pagneux et al., 2020). To achieve this goal, hyaluronic acid dissolving microneedle (MN) arrays were developed utilizing gold nanocages loaded with DOX (Figures 10D–F) (Dong et al., 2018). The fabricated MNs effectively penetrate the skin, dissolve, release drugs within the tumor, and exhibit photothermal and synergistic effects by near-infrared laser irradiation. The confocal laser scanning microscope (CLSM) provides a visual view of the release of gold nanocages perpendicular to the surface of the mouse skin (Figure 10E). In addition, red fluorescent arrays of gold nanocage spots were clearly detected in the skin. Fluorescent signals were strongly maintained between 150 and 230 μm and then decreased slowly, and the red spot in the CLSM and the black spot in the light field image almost overlapped in the merged image. This indicates that the released gold nanocages are directly inside the insertion path and only a small fraction spread into the surrounding tissue. Figure 10F shows the high tumor growth inhibition effect of gold nanocages with the simultaneous application of NIR and DOX within tumors.

### Biomedical Imaging: Theranostic Application

In general, gold nanoparticles are potentially applicable to a wide variety of fields in optical imaging, cell imaging, ultrasound imaging, and positron emission tomography. Biomedical imaging is a non-invasive functional imaging technology that detects and monitors various human physiological and pathological conditions. Its use allows the early detection of tumors and other diseases, and guides precision treatment. Gold nanoparticles are well known for their ability to increase biological inactivity and spatial and temporal resolution for imaging (Singh et al., 2018; García-Álvarez et al., 2020; Si et al., 2021; Qambrani et al., 2021).

**FIGURE 11:**
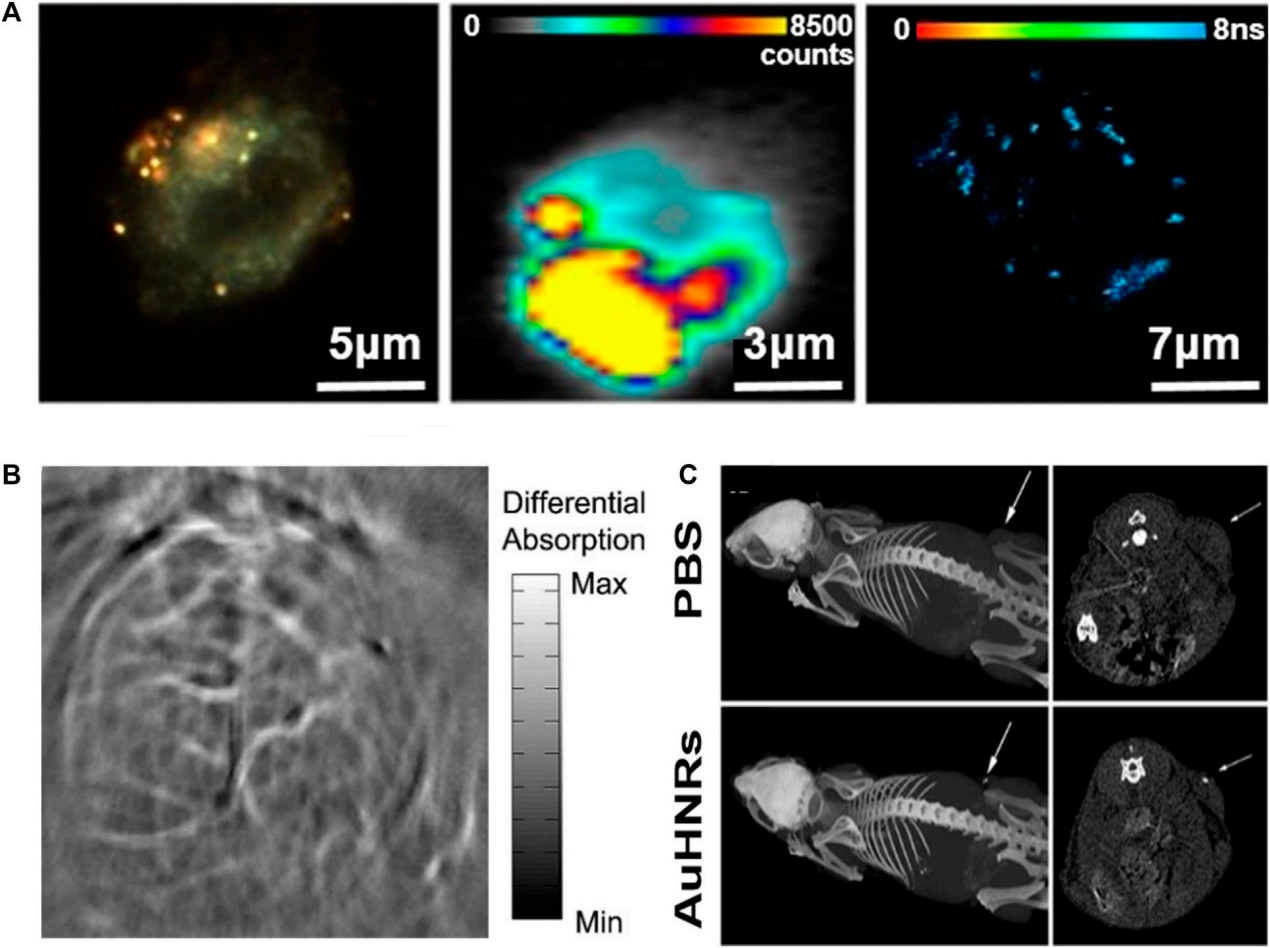
**(A)** Multimodal dark-field (left)/SERS (center)/two photon-FLIM imaging (right) of antibody-conjugated and Raman-tagged hollow gold nanospheres. Photoacoustic tomography (PAT) imaging of the mouse brain using gold nanostructures. **(B)** Non-invasive PAT imaging of a mouse brain *in vivo* employing polyethylene glycol (PEG)-hollow gold nanospheres and near-infrared (NIR) light at a wavelength of 800 nm. Hollow gold nanorods for bioimaging and combined chemo–photothermal therapy. **(C)** Computed tomography images of mice with an injection of phosphate-buffered saline and hollow gold nanorods. Arrows point to the tumor site. **(A)** Reprinted with permission from Nagy-Simon et al. (2017). Copyright 2017, American Chemical Society. **(B)** Reprinted with permission from Lu et al. (2010). Copyright 2009, Elsevier. **(C)** Reprinted with permission from Cai et al. (2018). Copyright 2018, American Chemical Society.

Hollow gold nanostructures can be used in various biomedical imaging fields, such as TEM, computed tomography, SERS imaging, and photoacoustic tomography (PAT). (Nagy-Simon et al., 2017) reported a new class of contrast medium containing NIR-reactive hollow gold nanospheres for the detection and multimodal imaging of CD19 (+) cancer lymphocytes (Figure 11A). The medium binds to an anti-CD19 monoclonal antibody based on hollow gold nanospheres and is denoted by the SERS active molecule Nile blue to complete the hollow gold nanosphere–Nile blue–PEG–anti-CD19 complex. TEM was used to investigate the specificity of this contrast medium, and dark-field, SERS, and two-photon excitation fluorescence lifetime imaging microscopy proved its interaction with TEM and the preferential internalization of the complex. Therefore, improvements in the SERS background occurred over the entire measurement interval and could only be observed in local areas corresponding to the accumulated nanoparticles. In conclusion, (Nagy-Simon et al., 2017) demonstrated that antibody complexes provide labeling molecules and SERS background signals for internalization and intracellular positioning, and are promising materials for non-invasive microscopic imaging.

PAT, also referred to as optoacoustic tomography or photoacoustic imaging, is a powerful nonionizing optical imaging modality that integrates the advantages of both optical radiation and ultrasonic detection (Yao and Wang, 2011; von Knorring and Mogensen, 2020). This imaging technology can be monitored with an optical contrast agent, such as gold, which facilitates high sensitivity and specificity, separating the signal contributions of several optical absorbers by spectroscopy, enabling simultaneous molecular and functional imaging. Recently, hollow gold nanospheres have emerged as novel molecular contrast agents for PAT; they can be adjusted to the near-infrared range, show strong resonance absorption and absorption peaks at ∼800 nm, and allow surface coating with thiolated polyethylene glycol (PEG), which leads to significantly higher PA efficiency (Lu et al., 2010). The PEGylated hollow gold nanospheres clearly expressed a small cerebral vascular structure with a diameter of ∼100 nm in PAT images obtained within 2 h of intravenous administration of nude mice (Figure 11B); however, preliminary results have not shown acute toxicity to the liver, spleen, or kidneys of mice, and consequently show promise as contrast agents for PAT with relatively high spatial resolution and improved sensitivity.

These bioimaging applications can be combined with therapeutic applications, such as drug delivery and PTT, to simultaneously perform treatment and diagnosis to enable precision medicine that can be tailored to each patient (Moorkoth et al., 2021; Yang et al., 2021). Nanoscale particles can be targeted to desired positions through surface functionality (Wijetunga et al., 2020). In particular, the utilization of hollow gold nanostructures can enhance the efficiency of theranostics owing to their inherent characteristics. For example, nontoxic hollow gold nanorods can be used as chemo–photothermal therapy substances at the same time and as contrast agents for multimodal imaging for cancer theranostics (Figure 11C) (Cai et al., 2018). The platform is a new type of nano-agent with LSPR peaks in the NIR-II window (1,000–1,350 nm), which is more suitable for biological applications owing to its deeper tissue penetration capabilities and higher maximum permissible exposure than that of the NIR-I window (700–950 nm). Figure 11C demonstrates the ability of the computed tomography imaging contrast agent by injecting hollow gold nanorods into a tumor-bearing mouse to confirm the brightness in the tumor region. After loading DOX, the laser was examined to confirm the chemo–photothermal effect. Although the data are not indicated in this paper, when phototherapy and chemotherapy were applied at the same time, the tumor disappeared completely after approximately 10 days, confirming the synergistic effect. Thus, it can be seen that hollow gold nanostructures show improved ability when applied with PTT as well as bioimaging.

## Conclusions and Future Perspectives

Hollow gold nanostructures have drawn attention for their biomedical applications because of their unique physical and optical properties, such as facile LSPR tunability, drug loading capacity, and enhanced photothermal activity, compared to those of conventional solid gold nanoparticles. In this review, we summarized the recent research trends in hollow gold nanostructures, including synthetic methods, physical/optical characteristics, and recent advances in biomedical applications. Various forms of hollow gold nanostructures can be synthesized by either hard- or soft-templating methods, but the majority of synthesis is based on galvanic replacement. While the LSPR peaks of solid gold nanoparticles depend solely on the overall size of the particles, those of hollow gold nanostructures are greatly influenced not only by the size, but also by the thickness of the shell. Therefore, the LSPR peaks can be easily adjusted while maintaining the overall size of the particles, which provides additional tunability of the SERS signal or photothermal activity. The voids in the interior also provide additional functionality to hollow gold nanostructures by being utilized as nanoscale storage for impregnating functional materials (e.g., anticancer drugs), which is not possible in solid gold nanoparticles. Based on these characteristics, various hollow gold nanostructures have been widely used in several biomedical applications, including biosensors, photothermal therapy, and biomedical imaging. It should be noted that there have been many studies aimed at the synergistic effects of simultaneously utilizing the various functionalities of hollow gold nanostructures, such as combined chemo–photothermal therapy, light-responsive drug release, and plasmonic nanostructure-based bioimaging.

Despite this progress, there are still many issues: 1) off- and on-target accumulation of hollow gold nanostructures and 2) preventing the aggregation and disassembly of hollow gold nanostructures. Large-sized hollow gold nanostructures (larger than 200 nm) can lead undesirable accumulation in the reticuloendothelial system (e.g., liver, spleen, and lungs); on the other hand, very small-sized one cannot provide sufficient on-target accumulation of them due to the short circulation time. Even if the size of these structures is suitable for selective accumulation, the aggregation and disassembly of hollow gold nanostructures in human body can decrease the LSPR intensity. Therefore, researchers should consider 1) appropriate size of nanostructures for sufficient on-target accumulation and prevention of off-target side effect of them; 2) antibody conjugation which enhance the capability of target adhesion; and 3) the stability of hollow gold nanostructures in human body to provide good LSPR properties. We strongly believe that the above-mentioned problems will be overcome by further research using advanced synthetic methods or surface modifications in the future; then, hollow gold nanostructures will become increasingly important in biomedical applications because it is clear that these unique structures have many advantages compared to conventional nanoparticles. We hope that this review will help readers to comprehend the recent advances in hollow gold nanostructures and will provide great inspiration for researchers.

## References

[B1] Aherne D. Gara M. Kelly J. M. Gun'ko Y. K. (2010). From Ag Nanoprisms to Triangular AuAg Nanoboxes. Adv. Funct. Mater. 20, 1329–1338. 10.1002/adfm.200902030

[B2] Azadmehr F. Zarei K. (2020). Ultrasensitive Determination of Ceftizoxime Using Pencil Graphite Electrode Modified by Hollow Gold Nanoparticles/reduced Graphene Oxide. Arabian J. Chem. 13, 1890–1900. 10.1016/j.arabjc.2018.02.004

[B3] Bao T. Yin W. Zheng X. Zhang X. Yu J. Dong X. (2016). One-pot Synthesis of PEGylated Plasmonic MoO3-X Hollow Nanospheres for Photoacoustic Imaging Guided Chemo-Photothermal Combinational Therapy of Cancer. Biomaterials 76, 11–24. 10.1016/j.biomaterials.2015.10.048 26517561

[B4] Bi J. Cai H. Wang B. Kong C. Yang S. (2019). Localized Surface Plasmon Enhanced Electrocatalytic Methanol Oxidation of AgPt Bimetallic Nanoparticles with an Ultra-thin Shell. Chem. Commun. 55, 3943–3946. 10.1039/C9CC00331B 30874259

[B5] Cai K. Zhang W. Foda M. F. Li X. Zhang J. Zhong Y. (2020a). Miniature Hollow Gold Nanorods with Enhanced Effect for *In Vivo* Photoacoustic Imaging in the NIR‐II Window. Small 16 (37), 2002748. 10.1002/smll.202002748 32780938

[B6] Cai K. Zhang W. Zhang J. Li H. Han H. Zhai T. (2018). Design of Gold Hollow Nanorods with Controllable Aspect Ratio for Multimodal Imaging and Combined Chemo-Photothermal Therapy in the Second Near-Infrared Window. ACS Appl. Mater. Inter. 10, 36703–36710. 10.1021/acsami.8b12758 30284807

[B7] Cai Z. Hu Y. Sun Y. Gu Q. Wu P. Cai C. (2020b). Plasmonic SERS Biosensor Based on Multibranched Gold Nanoparticles Embedded in Polydimethylsiloxane for Quantification of Hematin in Human Erythrocytes. Anal. Chem. 93, 1025–1032. 10.1021/acs.analchem.0c03921 33284601

[B8] Chen J. Mclellan J. M. Siekkinen A. Xiong Y. Li Z.-Y. Xia Y. (2006). Facile Synthesis of Gold−Silver Nanocages with Controllable Pores on the Surface. J. Am. Chem. Soc. 128, 14776–14777. 10.1021/ja066023g 17105266 PMC2532083

[B9] Chen M. Wu D. Tu S. Yang C. Chen D. Xu Y. (2021). A Novel Biosensor for the Ultrasensitive Detection of the lncRNA Biomarker MALAT1 in Non-small Cell Lung Cancer. Sci. Rep. 11, 1–11. 10.1038/s41598-021-83244-7 33574438 PMC7878801

[B10] Chithrani B. D. Ghazani A. A. Chan W. C. W. (2006). Determining the Size and Shape Dependence of Gold Nanoparticle Uptake into Mammalian Cells. Nano Lett. 6, 662–668. 10.1021/nl052396o 16608261

[B11] Choi N. Dang H. Das A. Sim M. S. Chung I. Y. Choo J. (2020). SERS Biosensors for Ultrasensitive Detection of Multiple Biomarkers Expressed in Cancer Cells. Biosens. Bioelectron. 164, 112326. 10.1016/j.bios.2020.112326 32553352

[B92] Chung E. Jeon J. Yu J. Lee C. Choo J. (2015). Surface-Enhanced Raman Scattering Aptasensor for Ultrasensitive Trace Analysis of Bisphenol. A. Biosens Bioelectron 64, 560–565. 10.1016/j.bios.2014.09.087 25310489

[B12] Cobley C. M. Xia Y. (2010). Engineering the Properties of Metal Nanostructures *via* Galvanic Replacement Reactions. Mater. Sci. Eng. R: Rep. 70, 44–62. 10.1016/j.mser.2010.06.002 21180400 PMC3003924

[B13] Conroy P. J. Hearty S. Leonard P. O’Kennedy R. J. (2009). Antibody Production, Design and Use for Biosensor-Based Applications. Semin. Cel Developmental Biol. 20, 10–26. 10.1016/j.semcdb.2009.01.010 19429487

[B14] Cui G. He P. Yu L. Wen C. Xie X. Yao G. (2020). Oxygen Self-Enriched Nanoplatform Combined with US Imaging and Chemo/photothermal Therapy for Breast Cancer. Nanomedicine: Nanotechnology, Biol. Med. 29, 102238. 10.1016/j.nano.2020.102238 32565228

[B15] Depciuch J. Stec M. Maximenko A. Baran J. Parlinska-Wojtan M. (2020). Temperature-controlled Synthesis of Hollow, Porous Gold Nanoparticles with Wide Range Light Absorption. J. Mater. Sci. 55 (12), 5257–5267. 10.1007/s10853-020-04345-8

[B16] Ding X. Liow C. H. Zhang M. Huang R. Li C. Shen H. (2014). Surface Plasmon Resonance Enhanced Light Absorption and Photothermal Therapy in the Second Near-Infrared Window. J. Am. Chem. Soc. 136, 15684–15693. 10.1021/ja508641z 25340966

[B17] Dong L. Li Y. Li Z. Xu N. Liu P. Du H. (2018). Au Nanocage-Strengthened Dissolving Microneedles for Chemo-Photothermal Combined Therapy of Superficial Skin Tumors. ACS Appl. Mater. Inter. 10, 9247–9256. 10.1021/acsami.7b18293 29493217

[B18] Fang X. Lui K.-H. Li S. Lo W.-S. Li X. Gu Y. (2020). Multifunctional Nanotheranostic Gold Nanocage/Selenium Core-Shell for PAI-Guided Chemo-Photothermal Synergistic Therapy *In Vivo* . Ijn Vol. 15, 10271–10284. 10.2147/IJN.S275846 PMC7751612 33364758

[B19] Farokhnezhad M. Esmaeilzadeh M. (2019). Graphene Coated Gold Nanoparticles: an Emerging Class of Nanoagents for Photothermal Therapy Applications. Phys. Chem. Chem. Phys. 21, 18352–18362. 10.1039/C9CP03126J 31402363

[B20] Feng A. L. You M. L. Tian L. Singamaneni S. Liu M. Duan Z. (2015). Distance-dependent Plasmon-Enhanced Fluorescence of Upconversion Nanoparticles Using Polyelectrolyte Multilayers as Tunable Spacers. Sci. Rep. 5, 1–10. 10.1038/srep07779 PMC5379003 25586238

[B21] Gan R. Fan H. Wei Z. Liu H. Lan S. Dai Q. (2019). Photothermal Response of Hollow Gold Nanorods under Femtosecond Laser Irradiation. Nanomaterials 9, 711. 10.3390/nano9050711 PMC6566344 31067807

[B22] Gao Y. Kang J. Lei Z. Li Y. Mei X. Wang G. (2020). Use of the Highly Biocompatible Au Nanocages@ PEG Nanoparticles as a New Contrast Agent for *In Vivo* Computed Tomography Scan Imaging. Nanoscale Res. Lett. 15 (1), 1–9. 10.1186/s11671-020-3286-2 32130549 PMC7056796

[B23] García-Álvarez R. Chen L. Nedilko A. Sánchez-Iglesias A. Rix A. Lederle W. (2020). Optimizing the Geometry of Photoacoustically Active Gold Nanoparticles for Biomedical Imaging. ACS Photon. 7, 646–652. 10.1021/acsphotonics.9b01418

[B24] Goode J. A. Rushworth J. V. H. Millner P. A. (2015). Biosensor Regeneration: a Review of Common Techniques and Outcomes. Langmuir 31, 6267–6276. 10.1021/la503533g 25402969

[B25] Goodman A. M. Cao Y. Urban C. Neumann O. Ayala-Orozco C. Knight M. W. (2014). The Surprising *In Vivo* Instability of Near-IR-Absorbing Hollow Au-Ag Nanoshells. ACS Nano 8, 3222–3231. 10.1021/nn405663h 24547810 PMC4004326

[B26] Grabowska-Jadach I. Kalinowska D. Drozd M. Pietrzak M. (2019). Synthesis, Characterization and Application of Plasmonic Hollow Gold Nanoshells in a Photothermal Therapy-New Particles for Theranostics. Biomed. Pharmacother. 111, 1147–1155. 10.1016/j.biopha.2019.01.037 30841428

[B27] Han J. Li J. Jia W. Yao L. Li X. Jiang L. (2014). Photothermal Therapy of Cancer Cells Using Novel Hollow Gold Nanoflowers. Int. J. Nanomedicine 9, 517–526. 10.2147/IJN.S55800 24549034 PMC3897319

[B28] Han S. Han K. Hong J. Yoon D.-Y. Park C. Kim Y. (2018). Photothermal Cellulose-Patch with Gold-Spiked Silica Microrods Based on *Escherichia coli* . ACS Omega 3, 5244–5251. 10.1021/acsomega.8b00639 30023911 PMC6045327

[B29] He J. Wei Q. Wang S. Hua S. Zhou M. (2021). Bioinspired Protein corona Strategy Enhanced Biocompatibility of Ag-Hybrid Hollow Au Nanoshells for Surface-Enhanced Raman Scattering Imaging and On-Demand Activation Tumor-Phototherapy. Biomaterials 271, 120734. 10.1016/j.biomaterials.2021.120734 33647873

[B30] Hong X. Wang D. Cai S. Rong H. Li Y. (2012). Single-Crystalline Octahedral Au-Ag Nanoframes. J. Am. Chem. Soc. 134, 18165–18168. 10.1021/ja3076132 23088493

[B31] Jensen T. R. Malinsky M. D. Haynes C. L. Van Duyne R. P. (2000). Nanosphere Lithography: Tunable Localized Surface Plasmon Resonance Spectra of Silver Nanoparticles. J. Phys. Chem. B 104, 10549–10556. 10.1021/jp002435e

[B32] Jeong S. Kim M.-W. Jo Y.-R. Kim N.-Y. Kang D. Lee S. Y. (2019). Hollow Porous Gold Nanoshells with Controlled Nanojunctions for Highly Tunable Plasmon Resonances and Intense Field Enhancements for Surface-Enhanced Raman Scattering. ACS Appl. Mater. Inter. 11, 44458–44465. 10.1021/acsami.9b16983 31718128

[B33] Kim J. K. Park T.-H. Jang D.-J. (2020). Surface-enhanced Raman Scattering and Photothermal Effect of Hollow Au Nanourchins with Well-Defined Cavities. J. Nanopart. Res. 22, 1–11. 10.1007/s11051-020-05034-y

[B34] Ko J. Lee C. Choo J. (2015). Highly Sensitive SERS-Based Immunoassay of Aflatoxin B1 Using Silica-Encapsulated Hollow Gold Nanoparticles. J. Hazard. Mater. 285, 11–17. 10.1016/j.jhazmat.2014.11.018 25462866

[B35] Ko J. Lee S. Lee E. K. Chang S.-I. Chen L. Yoon S.-Y. (2013). SERS-based Immunoassay of Tumor Marker VEGF Using DNA Aptamers and Silica-Encapsulated Hollow Gold Nanospheres. Phys. Chem. Chem. Phys. 15, 5379–5385. 10.1039/C2CP43155F 23201966

[B36] Kreibig U. Vollmer M. (2013). Optical Properties of Metal Clusters. Berlin: Springer-Verlag

[B37] Lee J. H. Jeong H. S. Lee D. H. Beack S. Kim T. Lee G.-H. (2017). Targeted Hyaluronate-Hollow Gold Nanosphere Conjugate for Anti-obesity Photothermal Lipolysis. ACS Biomater. Sci. Eng. 3, 3646–3653. 10.1021/acsbiomaterials.7b00549 33445399

[B38] Lee K. E. Hesketh A. V. Kelly T. L. (2014). Chemical Stability and Degradation Mechanisms of Triangular Ag, Ag@Au, and Au Nanoprisms. Phys. Chem. Chem. Phys. 16, 12407–12414. 10.1039/C4CP00954A 24827005

[B39] Lee S.-W. Lee K.-S. Ahn J. Lee J.-J. Kim M.-G. Shin Y.-B. (2011). Highly Sensitive Biosensing Using Arrays of Plasmonic Au Nanodisks Realized by Nanoimprint Lithography. ACS Nano 5, 897–904. 10.1021/nn102041m 21222487

[B40] Lee T. Kim G. H. Kim S. M. Hong K. Kim Y. Park C. (2019). Label-free Localized Surface Plasmon Resonance Biosensor Composed of Multi-Functional DNA 3 Way junction on Hollow Au Spike-like Nanoparticles (HAuSN) for Avian Influenza Virus Detection. Colloids Surf. B: Biointerfaces 182, 110341. 10.1016/j.colsurfb.2019.06.070 31284148 PMC7185628

[B41] Li C. Ye R. Bouckaert J. Zurutuza A. Drider D. Dumych T. (2017). Flexible Nanoholey Patches for Antibiotic-free Treatments of Skin Infections. ACS Appl. Mater. Inter. 9, 36665–36674. 10.1021/acsami.7b12949 28956593

[B42] Liang H.-P. Wan L.-J. Bai C.-L. Jiang L. (2005). Gold Hollow Nanospheres: Tunable Surface Plasmon Resonance Controlled by interior-cavity Sizes. J. Phys. Chem. B 109, 7795–7800. 10.1021/jp045006f 16851906

[B43] Lim D.-K. Jeon K.-S. Hwang J.-H. Kim H. Kwon S. Suh Y. D. (2011). Highly Uniform and Reproducible Surface-Enhanced Raman Scattering from DNA-Tailorable Nanoparticles with 1-nm interior gap. Nat. Nanotech 6, 452–460. 10.1038/nnano.2011.79 21623360

[B44] Lindley S. A. Cooper J. K. Rojas-Andrade M. D. Fung V. Leahy C. J. Chen S. (2018). Highly Tunable Hollow Gold Nanospheres: Gaining Size Control and Uniform Galvanic Exchange of Sacrificial Cobalt Boride Scaffolds. ACS Appl. Mater. Inter. 10, 12992–13001. 10.1021/acsami.8b00726 29624054

[B45] Lindley S. A. Zhang J. Z. (2019). Bumpy Hollow Gold Nanospheres for Theranostic Applications: Effect of Surface Morphology on Photothermal Conversion Efficiency. ACS Appl. Nano Mater. 2, 1072–1081. 10.1021/acsanm.8b02331

[B46] Ling C. Wang X. Shen Y. (2021). Advances in Hollow Inorganic Nanomedicines for Photothermal-Based Therapies. Ijn Vol. 16, 493–513. 10.2147/IJN.S285115 PMC7837554 33519198

[B93] Lopez-Chaves C. Soto-Alvaredo J. Montes-Bayon M. Bettmer J. Llopis J. Sanchez-Gonzalez C. (2018). Gold Nanoparticles: Distribution, Bioaccumulation and Toxicity. In Vitro and In Vivo Studies. Nanomedicine 14 (1), 1–12. 10.1016/j.nano.2017.08.011 28882675

[B47] Lu W. Huang Q. Ku G. Wen X. Zhou M. Guzatov D. (2010). Photoacoustic Imaging of Living Mouse Brain Vasculature Using Hollow Gold Nanospheres. Biomaterials 31, 2617–2626. 10.1016/j.biomaterials.2009.12.007 20036000 PMC2813997

[B48] Lu X. Au L. Mclellan J. Li Z.-Y. Marquez M. Xia Y. (2007a). Fabrication of Cubic Nanocages and Nanoframes by Dealloying Au/Ag Alloy Nanoboxes with an Aqueous Etchant Based on Fe(NO3)3 or NH4OH. Nano Lett. 7, 1764–1769. 10.1021/nl070838l 17489641 PMC2504472

[B49] Lu X. Chen J. Skrabalak S. E. Xia Y. (2007b). Galvanic Replacement Reaction: A Simple and Powerful Route to Hollow and Porous Metal Nanostructures. Proc. Inst. Mech. Eng. N: J. Nanoengineering Nanosystems 221, 1–16. 10.1243/17403499JNN111

[B50] Maier S. A. (2007). Plasmonics: Fundamentals and Applications. New York: Springer.

[B51] Mayer K. M. Hao F. Lee S. Nordlander P. Hafner J. H. (2010). A Single Molecule Immunoassay by Localized Surface Plasmon Resonance. Nanotechnology 21, 255503. 10.1088/0957-4484/21/25/255503 20516579

[B52] Meeker D. G. Jenkins S. V. Miller E. K. Beenken K. E. Loughran A. J. Powless A. (2016). Synergistic Photothermal and Antibiotic Killing of Biofilm-Associated *Staphylococcus aureus* Using Targeted Antibiotic-Loaded Gold Nanoconstructs. ACS Infect. Dis. 2, 241–250. 10.1021/acsinfecdis.5b00117 27441208 PMC4945994

[B53] Moorkoth D. Nampoothiri K. M. Nagarajan S. Ravindran Girija A. Balasubramaniyan S. Kumar D. S. (2021). Star-shaped Polylactide Dipyridamole Conjugated to 5-fluorouracil and 4-piperidinopiperidine Nanocarriers for Bioimaging and Dual Drug Delivery in Cancer Cells. ACS Appl. Polym. Mater. 3, 737–756. 10.1021/acsapm.0c01043

[B54] Nagy-Simon T. Tatar A.-S. Craciun A.-M. Vulpoi A. Jurj M.-A. Florea A. (2017). Antibody Conjugated, Raman Tagged Hollow Gold-Silver Nanospheres for Specific Targeting and Multimodal Dark-Field/SERS/Two Photon-FLIM Imaging of CD19(+) B Lymphoblasts. ACS Appl. Mater. Inter. 9, 21155–21168. 10.1021/acsami.7b05145 28574250

[B55] Oh M. H. Yu T. Yu S.-H. Lim B. Ko K.-T. Willinger M.-G. (2013). Galvanic Replacement Reactions in Metal Oxide Nanocrystals. Science 340, 964–968. 10.1126/science.1234751 23704569

[B56] Pagneux Q. Ye R. Chengnan L. Barras A. Hennuyer N. Staels B. (2020). Electrothermal Patches Driving the Transdermal Delivery of Insulin. Nanoscale Horiz. 5, 663–670. 10.1039/c9nh00576e 32226966

[B57] Prieto M. Arenal R. Henrard L. Gomez L. Sebastian V. Arruebo M. (2014). Morphological Tunability of the Plasmonic Response: from Hollow Gold Nanoparticles to Gold Nanorings. J. Phys. Chem. C 118, 28804–28811. 10.1021/jp5096129

[B58] Prodan E. Radloff C. Halas N. J. Nordlander P. (2003). A Hybridization Model for the Plasmon Response of Complex Nanostructures. Science 302, 419–422. 10.1126/science.1089171 14564001

[B59] Pu Y.-C. Song F. Zhang W. Lindley S. Adams S. Zhang J. Z. (2017). Size-Tunable Synthesis of Hollow Gold Nanospheres through Control of Reaction Temperature. Part. Part. Syst. Charact. 34, 1600255. 10.1002/ppsc.201600255

[B60] Qambrani A. Rehman F. U. Tanziela T. Shaikh S. Semcheddine F. Du T. (2021). Biocompatible Exosomes Nanodrug Cargo for Cancer Cell Bioimaging and Drug Delivery. Biomed. Mater. 16, 025026. 10.1088/1748-605X/abaaa2 32726764

[B61] Ren Q.-Q. Bai L.-Y. Zhang X.-S. Ma Z.-Y. Liu B. Zhao Y.-D. (2015). Preparation, Modification, and Application of Hollow Gold Nanospheres. J. Nanomater 2015, 1–7. 10.1155/2015/534070

[B62] Satija J. Tharion J. Mukherji S. (2015). Facile Synthesis of Size and Wavelength Tunable Hollow Gold Nanostructures for the Development of a LSPR Based Label-free Fiber-Optic Biosensor. RSC Adv. 5, 69970–69979. 10.1039/C5RA13941D

[B63] Sekhon J. S. (2021). Facile Tuning and Refractive Index Sensitivity of Localized Surface Plasmon Resonance Inflection Points in Hollow Silver Nanoshells. Plasmonics 16, 283–292. 10.1007/s11468-020-01277-6

[B64] Shen Y. Xia Y. Yang E. Ye Z. Ding Y. Tu J. (2020). A Polyoxyethylene Sorbitan Oleate Modified Hollow Gold Nanoparticle System to Escape Macrophage Phagocytosis Designed for Triple Combination Lung Cancer Therapy *via* LDL-R Mediated Endocytosis. Drug Deliv. 27, 1342–1359. 10.1080/10717544.2020.1822459 32964732 PMC7534200

[B65] Si P. Razmi N. Nur O. Solanki S. Pandey C. M. Gupta R. K. (2021). Gold Nanomaterials for Optical Biosensing and Bioimaging. Nanoscale Adv. 3 (10), 2679–2698. 10.1039/d0na00961 PMC9418567 36134176

[B66] Singh P. Pandit S. Mokkapati V. R. S. S. Garg A. Ravikumar V. Mijakovic I. (2018). Gold Nanoparticles in Diagnostics and Therapeutics for Human Cancer. Ijms 19, 1979. 10.3390/ijms19071979 PMC6073740 29986450

[B67] Skrabalak S. E. Au L. Li X. Xia Y. (2007). Facile Synthesis of Ag Nanocubes and Au Nanocages. Nat. Protoc. 2, 2182–2190. 10.1038/nprot.2007.326 17853874

[B68] Skrabalak S. E. Chen J. Sun Y. Lu X. Au L. Cobley C. M. (2008). Gold Nanocages: Synthesis, Properties, and Applications. Acc. Chem. Res. 41, 1587–1595. 10.1021/ar800018v 18570442 PMC2645935

[B69] Sun H. Su J. Meng Q. Yin Q. Chen L. Gu W. (2017). Cancer Cell Membrane-Coated Gold Nanocages with Hyperthermia-Triggered Drug Release and Homotypic Target Inhibit Growth and Metastasis of Breast Cancer. Adv. Funct. Mater. 27, 1604300. 10.1002/adfm.201604300

[B70] Sun X. He G. Xiong C. Wang C. Lian X. Hu L. (2021). One-Pot Fabrication of Hollow Porphyrinic MOF Nanoparticles with Ultrahigh Drug Loading toward Controlled Delivery and Synergistic Cancer Therapy. ACS Appl. Mater. Inter. 13, 3679–3693. 10.1021/acsami.0c20617 33464038

[B71] Sun Y. Li T. (2018). Composition-tunable Hollow Au/Ag SERS Nanoprobes Coupled with Target-Catalyzed Hairpin Assembly for Triple-Amplification Detection of miRNA. Anal. Chem. 90, 11614–11621. 10.1021/acs.analchem.8b03067 30175580

[B72] Sun Y. Xia Y. (2004). Mechanistic Study on the Replacement Reaction between Silver Nanostructures and Chloroauric Acid in Aqueous Medium. J. Am. Chem. Soc. 126, 3892–3901. 10.1021/ja039734c 15038743

[B73] Sutter E. Zhang B. Sutter P. (2020). DNA-mediated Three-Dimensional Assembly of Hollow Au-Ag Alloy Nanocages as Plasmonic Crystals. ACS Appl. Nano Mater. 3, 8068–8074. 10.1021/acsanm.0c01528

[B74] Tiwari P. Vig K. Dennis V. Singh S. (2011). Functionalized Gold Nanoparticles and Their Biomedical Applications. Nanomaterials 1, 31–63. 10.3390/nano1010031 28348279 PMC5315048

[B75] Tran N. T. Liao H. Feng X. Xu Z. Z. Liedberg B. (2020). Synthesis of Highly Branched Hollow Trimetallic PdAgCu Nanoparticles. Nanotechnology 31, 185601. 10.1088/1361-6528/ab6d25 31952066

[B76] Von Knorring T. Mogensen M. (2020). Photoacoustic Tomography for Assessment and Quantification of Cutaneous and Metastatic Malignant Melanoma – A Systematic Review. Photodiagnosis Photodyn Ther. 33, 102095. 10.1016/j.pdpdt.2020.102095 33188938

[B77] Wang H. Han J. Lu W. Zhang J. Li J. Jiang L. (2015a). Facile Preparation of Gold Nanocages and Hollow Gold Nanospheres *via* Solvent thermal Treatment and Their Surface Plasmon Resonance and Photothermal Properties. J. Colloid Interf. Sci. 440, 236–244. 10.1016/j.jcis.2014.11.004 25460711

[B78] Wang M. Yang Q. Li M. Zou H. Wang Z. Ran H. (2020). Multifunctional Nanoparticles for Multimodal Imaging-Guided Low-Intensity Focused Ultrasound/Immunosynergistic Retinoblastoma Therapy. ACS Appl. Mater. Inter. 12 (5), 5642–5657. 10.1021/acsami.9b22072 31940169

[B79] Wang W. Han Y. Tian M. Fan Y. Tang Y. Gao M. (2013). Cationic Gemini Surfactant-Assisted Synthesis of Hollow Au Nanostructures by Stepwise Reductions. ACS Appl. Mater. Inter. 5, 5709–5716. 10.1021/am4011226 23725038

[B80] Wang W. Zhao N. Li X. Wan J. Luo X. (2015b). Isothermal Amplified Detection of ATP Using Au Nanocages Capped with a DNA Molecular Gate and its Application in Cell Lysates. Analyst 140, 1672–1677. 10.1039/C4AN02202E 25627025

[B81] Wang Y. Zhao P. Mao L. Hou Y. Li D. (2018). Determination of Brain Injury Biomarkers by Surface-Enhanced Raman Scattering Using Hollow Gold Nanospheres. RSC Adv. 8, 3143–3150. 10.1039/C7RA12410D PMC9077554 35541182

[B82] Wijetunga I. Mcveigh L. E. Charalambous A. Antanaviciute A. Carr I. M. Nair A. (2020). Translating Biomarkers of Cholangiocarcinoma for Theranosis: A Systematic Review. Cancers 12, 2817. 10.3390/cancers12102817 PMC7601719 33007872

[B83] Xia X. Wang Y. Ruditskiy A. Xia Y. (2013). 25th Anniversary Article: Galvanic Replacement: a Simple and Versatile Route to Hollow Nanostructures with Tunable and Well-Controlled Properties. Adv. Mater. 25, 6313–6333. 10.1002/adma.201302820 24027074

[B94] Xu P. Wang R. Yang W. Liu Y. He D. Ye Z. (2021). A DM1-Doped Porous Gold Nanoshell System for NIR Accelerated Redox-Responsive Release and Triple Modal Imaging Guided Photothermal Synergistic Chemotherapy. J. Nanobiotechnol. 19 (1), 1–19. 10.1186/s12951-021-00824-5 PMC7976706 33741008

[B84] Yang M. Deng J. Su H. Gu S. Zhang J. Zhong A. (2021). Small Organic Molecule-Based Nanoparticles with Red/near-Infrared Aggregation-Induced Emission for Bioimaging and PDT/PTT Synergistic Therapy. Mater. Chem. Front. 5, 406–417. 10.1039/D0QM00536C

[B85] Yao J. Wang L. V. (2011). Photoacoustic Tomography: Fundamentals, Advances and Prospects. Contrast Media Mol. Imaging 6, 332–345. 10.1002/cmmi.443 22025335 PMC3205414

[B86] Ye P. Xin W. De Rosa I. M. Wang Y. Goorsky M. S. Zheng L. (2020). One-pot Self-Templated Growth of Gold Nanoframes for Enhanced Surface-Enhanced Raman Scattering Performance. ACS Appl. Mater. Inter. 12, 22050–22057. 10.1021/acsami.0c04777 32266808

[B87] Yoon J. Choi N. Ko J. Kim K. Lee S. Choo J. (2013). Highly Sensitive Detection of Thrombin Using SERS-Based Magnetic Aptasensors. Biosens. Bioelectron. 42, 62–67. 10.1016/j.bios.2013.03.003 23557978

[B96] Zeng L. Li Y. Guo L. Wang Z. Xu X. (2020). Rapid, Ultrasensitive and Highly Specific Biosensor for the Diagnosis of SARS-CoV-2 in Clinical Blood Samples. Mater. Chem. Front. 4 (7), 2000–2005. 10.1039/D0QM00294A

[B88] Zhang W. Cai K. Li X. Zhang J. Ma Z. Foda M. F. (2019). Au Hollow Nanorods-Chimeric Peptide Nanocarrier for NIR-II Photothermal Therapy and Real-Time Apoptosis Imaging for Tumor Theranostics. Theranostics 9, 4971–4981. 10.7150/thno.35560 31410195 PMC6691385

[B89] Zhang W. Yang J. Lu X. (2012). Tailoring Galvanic Replacement Reaction for the Preparation of Pt/Ag Bimetallic Hollow Nanostructures with Controlled Number of Voids. ACS Nano 6, 7397–7405. 10.1021/nn302590k 22804563

[B90] Zhang Z. Wang H. Chen Z. Wang X. Choo J. Chen L. (2018). Plasmonic Colorimetric Sensors Based on Etching and Growth of noble Metal Nanoparticles: Strategies and Applications. Biosens. Bioelectron. 114, 52–65. 10.1016/j.bios.2018.05.015 29778002

[B91] Zhu D. Liu Y. Liu M. Liu X. Prasad P. N. Swihart M. T. (2020). Galvanic Replacement Synthesis of Multi-Branched Gold Nanocrystals for Photothermal Cancer Therapy. J. Mater. Chem. B 8, 5491–5499. 10.1039/D0TB00748J 32478780

